# A preliminary approach to quantifying the overall environmental risks posed by development projects during environmental impact assessment

**DOI:** 10.1371/journal.pone.0180982

**Published:** 2017-07-07

**Authors:** Sam Nicol, Iadine Chadès

**Affiliations:** CSIRO Land and Water, Dutton Park, Queensland, Australia; Southwest University, CHINA

## Abstract

Environmental impact assessment (EIA) is used globally to manage the impacts of development projects on the environment, so there is an imperative to demonstrate that it can effectively identify risky projects. However, despite the widespread use of quantitative predictive risk models in areas such as toxicology, ecosystem modelling and water quality, the use of predictive risk tools to assess the overall expected environmental impacts of major construction and development proposals is comparatively rare. A risk-based approach has many potential advantages, including improved prediction and attribution of cause and effect; sensitivity analysis; continual learning; and optimal resource allocation. In this paper we investigate the feasibility of using a Bayesian belief network (BBN) to quantify the likelihood and consequence of non-compliance of new projects based on the occurrence probabilities of a set of expert-defined features. The BBN incorporates expert knowledge and continually improves its predictions based on new data as it is collected. We use simulation to explore the trade-off between the number of data points and the prediction accuracy of the BBN, and find that the BBN could predict risk with 90% accuracy using approximately 1000 data points. Although a further pilot test with real project data is required, our results suggest that a BBN is a promising method to monitor overall risks posed by development within an existing EIA process given a modest investment in data collection.

## Introduction

In many countries, environmental impact assessment (EIA) is the main vehicle to protect the environment [1]. For the purposes of this paper, ‘EIA’ refers specifically to the assessment process that considers the potential impacts posed by proposed construction and development projects that will impact environmental features that are legally protected by environmental law. Where impacts of development projects will occur, the main opportunity for prevention and mitigation occurs during EIA, and the conditions that define a future breach of the law are set during EIA. It is therefore critical that the EIA process is able to correctly predict the overall risk (herafter ‘risk’) posed by development, i.e., the likelihood and consequence that a project will have an impact on the environment.

Many development projects proceed in accordance with the conditions set during the EIA process. However some approved development projects experience operational failures which, in worst case scenarios, go on to cause severe damage to the environment. For example, the environmental impacts of the 2010 Deepwater Horizon oil spill in the United States [2] made world headlines; among the impacts was a predicted cost of US$8.7 billion to fisheries alone [3]. These failures can be devastating to the environment and the local communities, embarrassing for the regulator, and expensive or impossible to remediate for proponents.

Strong regulatory oversight of development projects that require EIA should in principle identify likely, high consequence environmental impacts early and prevent breaches of environmental conditions after approval is granted. However, limited resources for environment regulation and compliance monitoring mean that resources must be directed to where they are most needed [4–8]. A result of limited resources is that resources are often directed to high-profile, high-budget, or politically sensitive projects [8]. However this approach may not necessarily be the best use of resources, as there may exist other projects that pose higher risks of causing environmental harm. A rational prioritisation tool may help to direct resources to projects that are most likely to cause significant environmental harm and improve the effectiveness of the EIA process [4, 9].

Quantitative risk assessment is the norm in many environmental disciplines, including toxicology [10], water quality [11] and ecosystem modelling [12, 13], and some of these disciplines routinely carry out impact assessment for discipline-specific purposes. In this paper we limit our definition of ‘EIA’ to the statutory process of EIA as it is used for assessing the overall expected environmental impact of major development projects (e.g. infrastructure construction and mine creation) [14]. Unlike the quantitative EIA studies of (for example) toxicology or water quality, these development EIA studies tend to be descriptive and focused on procedural requirements [15]. While there is no real impediment to using a risk-based approach to EIA for development projects [16], much of the research into EIA has focused on the theory, practice and review of EIA [1]. Less attention has been given to methods to aid prediction in real systems and projects, and many agencies continue to apply descriptive EIA methods when assessing development projects.

We worked with the Australian Government Department of the Environment (DoE) to implement a structured risk management framework applicable to the assessment and compliance stages of the EIA process [17, 18], as part of an ongoing process to improve the DoE’s capacity to assess and manage risk [6]. The DoE designs and implements the policies and programmes to protect and conserve the environment and regulates the protection of Matters of National Environmental Significance (MNES), as described in the *Environment Protection and Biodiversity Conservation Act* [14]; this includes the responsibility to implement EIA for projects affecting MNES; see also S1 File. Because the DoE do not have a historical database of all of the risk factors recommended by this study in an accessible form, generating a risk tool will require a significant investment into data collection by the DoE. An additional project goal was to justify future data collection by determining the feasibility of a risk-based approach and quantifying the amount of data required to obtain output that was sufficiently accurate to be reliably used.

Our approach is divided into two parts. In the first iteration of our project, we implemented a weighted-sum risk tool that measured the risk posed by various indicators, and combined them using weights that specified the perceived relative importance of each indicator to obtain an overall risk score. Although the weighted-sum approach is simple and commonly used in risk assessment to prioritise projects, it is designed to represent decision makers’ attitudes to multiple risk factors rather than to quantify the risk of adverse outcomes. This means that weighted-sum methods have a number of drawbacks when used for risk assessment. Firstly, weighted-sum approaches have no causal model behind them, so it is hard to interpret the resulting scores. This does not matter when prioritising a set of projects (the relative scores are sufficient to rank projects); however when assessing the risk of a single project such as a new project proposal, it is important to be able to interpret the output of the risk score on a meaningful scale, such as a probability of failure [19]. Secondly, because the weights are additive, the approach implicitly assumes that risks are independent, which can lead to over- or under-estimation of risk if risk factors are correlated [19]. A better approach is to specify the relationships between variables and use the laws of probability to determine the chance that events occur simultaneously. Finally, the weighted-sum method has static subjective weights that are derived from DoE staff. Although expert information is often the only choice when decisions need to be made with limited data [20, 21], eliciting information from experts is known to suffer from a number of biases [22–24]. Best-practice risk management allows for continual improvement; this requires that data can be incorporated as it is collected and used as feedback to progressively improve prediction. This is not easily achieved with static weights.

To overcome the issues with weighted-sum methods, we demonstrate a novel technique to convert the commonly-used weighted-sum risk measure to a Bayesian belief network (BBN). A BBN is a graphical depiction of a risk process that represents causal dependencies between risk factors as probabilities [19]. Unlike weighted-sum methods, BBNs allow for relationships and correlations between indicators by requiring the modeller to specify causal models, and can learn from data rather than relying on value-laden weights that are static over time. BBNs have been used to develop decision support systems in a wide range of application domains including medical diagnosis [25, 26], safety assessment and fault diagnosis [27, 28], insurance and other financial fraud detection [29, 30], legal decisions [31, 32], forensics [33, 34] and ecology [12, 13, 35].

We use the BBN to explore the feasibility of quantifying the overall environmental risk posed by a proposed development application, with the goal of identifying high-risk proposals early to ensure that they receive an appropriate level of scrutiny during the EIA process. As well as illustrating the value of using a risk–based tool such as a BBN to predict development project risk, we show how to use simulated data to test the feasibility of a BBN prior to implementation by relating the predictive power of a BBN to the number of data points required.

## Methods

### Initial risk tool: A weighted-sum risk calculator

The DoE needed an easy-to-use, rapid prototype that would enable them to assess risk within their organisation and prioritise projects given their limited existing data and capacity for risk assessment. To rapidly obtain a workable prototype, we generated a weighted-sum risk calculator. This process required four key steps: defining risk, selecting risk factors or indicators, assigning indicator weights, and combining weights and indicators to obtain an overall risk score.

Risk is defined as the product of the likelihood and consequence of an event [36]. Likelihood was defined as the probability that the project will not be implemented in accordance with the approval document and fail to comply with associated conditions. Consequence was defined as a measure of the potential adverse impact on MNES, given the impact management strategies proposed.

After defining risk, the next stage of the project required selecting risk factors that were expected to lead to future non-compliance. Risk factors (indicators) were nominated by DoE staff who regularly work in EIA and compliance, drawing on the experiences of project officers and managers. Indicators were classified as either ‘likelihood’ or ‘consequence’ indicators. Each indicator was refined using the following criteria:

- Realistic/accessible—indicators were selected by DoE staff, with a criterion that they had to be easily accessible from existing data that were readily available to staff. To minimize the time required from staff to enter data, indicators with binary (yes/no) responses or simple categorical responses were preferred;
- Useful/relevant—indicators were selected using the experience of DoE staff, who are best placed to decide what indicators are relevant to risk;
- Quantitative/measurable—indicators had to be able to be converted to a number to facilitate aggregation into overall project risk. Responses that were potentially ambiguous should be avoided, however where ambiguity could exist, standard operating procedures would be written to minimise potential judgement inconsistencies; and
- Where relevant and not already considered, the indicators were also checked against the SMART criteria (i.e. specific, measurable, achievable, relevant and time-bound) often advocated in the literature (e.g. Niemeijer and de Groot ([37], Sommer, Zucca [38]).

The relative importance of indicators were weighted by DoE staff using a 0–100 scale. Staff were instructed to assign 100 points amongst the indicators, with higher scores representing indicators that were more influential in determining likelihood or consequence. Weights were elicited from a group of 10 staff representing different assessment and compliance divisions in a series of email and face-to-face interactions following a two-step Delphi method [24]. Participants in the elicitation were first asked to assign weights individually via email. Weights were finalised as a group in a workshop setting, where staff were able to discuss concerns and argue their ideas before forming a consensus. The group elected to select weights by forming a group consensus rather than by mathematical aggregation of individual weights, so point estimates of weights were obtained rather than distributions. The selected weights were further revised based on the results of an informal participatory process involving reviewing a set of trial projects with DoE staff. This process involved asking the DoE staff to assess the risk of small set of well-known projects using the weights and indicators, then using the participants’ knowledge of the projects to assess and revise the indicators and weights based on the order of the projects ranked by risk. A final set of indicators was selected after incorporating staff feedback (S2 File). Once the weights were agreed, likelihood and consequence of project non-compliance were calculated using a weighted-sum of the indicators, and risk was calculated using the product of likelihood and consequence [36]. Further details of indicator selection and weighting are contained in Nicol, Chades [17].

### Improving the prototype: Building Bayesian belief networks

Although the weighted-sum calculator met the immediate needs of the DoE by rapidly providing a simple tool to prioritise risk, weighted-sum methods suffer from a number of drawbacks (see Introduction). A solution to the issues with weighted-sum risk tools is to use a Bayesian Belief Network (BBN) as a risk model. Although methods exist for eliciting the data required for BBNs directly [39–41], this requires a substantial investment of engagement time with experts, which was not available in our study after the initial weighted-sum model had been developed. Instead, we used a novel method to convert the weighted-sum risk tool into an equivalent BBN. In this section we outline the BBN and how it was generated from the weighted-sum risk calculator.

In the case of predicting the risk of environmental non-compliance, a BBN appears as diagram showing a set of nodes that represent causal factors (i.e. risk indicators) and their relationship to a risk outcome. Nodes are linked by arrows which show the direction of causality (i.e. risk outcomes are caused by positive outcomes in risk indicators). Each indicator value and risk outcome is specified as a probability. Behind the visual depiction of the network are probability tables that quantify the relationships between linked risk factors and outcomes. The mechanics of a BBN are controlled by the laws of probability, particularly Bayes’ law, which specifies how to obtain the probability of an outcome based on causal conditions [19]. For a set of risk factors or indicators, the network can infer the likelihood of risk outcomes as well as quantify the relative contributions of each risk factor to an observed outcome.

We translated the weighted-sum risk calculator into a BBN framework using the program Netica, with a node for weights and equations at each node to specify the relationships between weights and risk (we follow the method described in Fenton and Neil (19], section 8.4.6; weighted-sum BBNs are included in S2 and S4 Files). In this approach, all risk factors from the weighted-sum model are included in the BBN as nodes with links to a risk output node (i.e. likelihood or consequence). An additional node is created to represent the weights; this node has one state for each risk factor, with assigned probability equal to the weight of that risk factor. The conditional probability table for the risk output node requires a partitioned equation that is conditioned on the weights node. To illustrate this, imagine a weighted-sum model with two risk factors, A and B, and risk output R. To create a BBN from this model, we create nodes for A and B with states ‘Yes’ and ‘No’ to indicate whether or not these risk factors were present. We also create a node ‘Weights’ with states W_A_ and W_B_; the states are assigned probabilities equivalent to the weights of risk factors A and B respectively. Finally, we create a risk output node R, with states ‘Yes’ (representing the probability that the risk is realised) and ‘No’ (probability that the risk is not realised). All nodes are linked to node R. For this model, the Netica equation for the risk output node would be:

$$\text{P}\left(\text{R}|\text{ Weights, A, B}\right)=\;\begin{array}{l} \text{Weights}={\text{ W}}_{\text{A}}\;?\;\left(\text{A}=\text{ Yes }?\;1:0\right)\;:\;\\ \text{Weights}={\text{ W}}_{\text{B}}\;?\;\left(\text{B}=\text{ Yes }?\;1:0\right)\;:\;0 \end{array}$$

In words, the first line of the right hand side of the equation reads: “if factor A is present, assign a score of W_A_ to R; if A is not present, then assign a contribution of 0 to R from factor A”. The second line is interpreted in the same way for risk factor B, and also stipulates that if neither factor is present, then the probability of R is zero. Further information on Netica’s equation syntax is included in the help files [42]; see also the instructions for this process included in S3 File.

**Fig 1:**
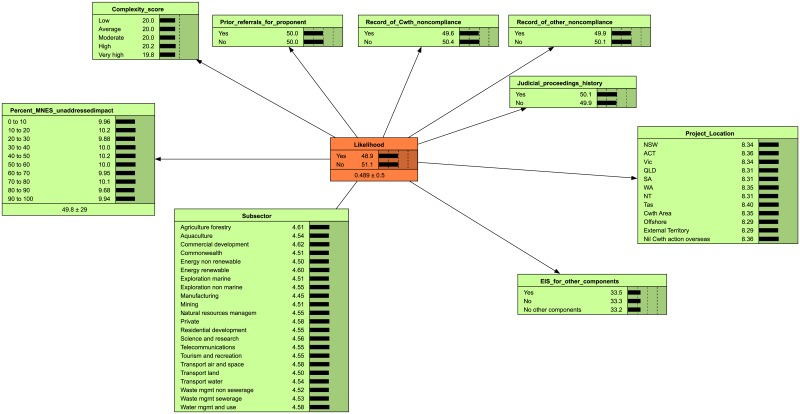
Likelihood naive BBN. Indicators (green nodes) influence the risk outcome (orange Likelihood node). Prior information for each node was learned from the weighted BBN. In the absence of any findings, the likelihood of an adverse outcome is 48.9%.

The approach outlined in the previous paragraph converts the weighted-sum model into a BBN. However because weights cannot be observed directly, they do not evolve in response to new data as it is gathered. To obtain a BBN that evolves in response to data while also retaining the prior information obtained from DoE staff, we had to eliminate the weights node so that all nodes were observable and could be updated with new information. To do this, we used a simulation approach to convert the weighted BBN to an equivalent unweighted naïve BBN [43] (Fig 1; see next paragraph and S3 File for details of the conversion process). Naïve BBNs simplify the conditional independence relationships between risk factors by assuming that risk factors are independent [43]. This also means that they have fewer parameters that need to be elicited from experts than more complex BBNs [43]. In many cases naïve BBNs have been shown to perform well even in cases when this assumption has not been proven [44].

The likelihood naïve BBN was built to perform in the same way as the weighted BBN. The principle of this approach is that if we sample enough case data (i.e. combinations of risk factors and risk outcomes) from the weighted-sum BBN, then we can use the case data to build a BBN that performs in the same way as the weighted-sum BBN but has a different structure (i.e. without the unobservable weighting node). We replicated the weighted likelihood BBN as a naïve BBN in Netica by simulating data (100,000 cases, obtained using Netica’s inbuilt “Simulate Cases” tool) using the distributions from the weighted BBN, then training a naïve BBN model using the cases. Because the naïve likelihood BBN is trained using data drawn from the weighted BBN (learning from data is carried out using Netica’s inbuilt “Learn->Incorp Case File” option), it predicts the same risk outcome as the weighted BBN but does not require a node for indicator weight (see S3 File for step-by-step details of this process).

**Fig 2:**
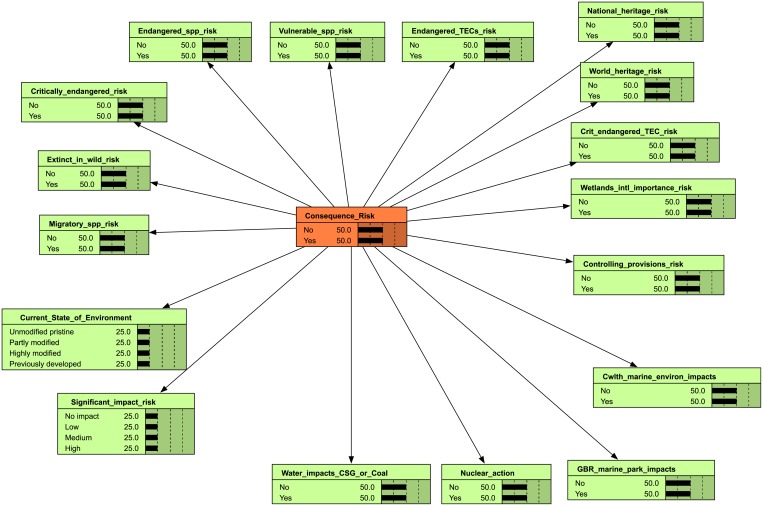
Consequence naive BBN. Indicators (green nodes) influence the risk outcome (orange Consequence node). Because the weighted consequence BBN could not be directly transformed into a naïve BBN, we built the naïve consequence BBN with no prior information.

We were unable to convert the weighted consequence BBN to a naïve model because the weighting calculation for the consequence model was complex (MNES risk was capped at a maximum score, which imposes a fixed value to each MNES that could not be learned from data). We proposed a simpler structure for the consequence naïve BBN that had fewer categories (Fig 2). This means that the consequence naïve BBN does not contain the prior weights, but it is a simpler network and will be able to learn from data as it becomes available. The final set of indicator questions used to construct the naïve likelihood and consequence BBNs are contained in Table 1.

**Table 1:** List of indicator questions used to determine project risk in the naive BBNs.

Indicator question	BBN Node Label	Possible scores
**Likelihood of project non-compliance**		
What is the relative complexity score based on total fee estimate? ^(1)^	Complexity_score	Discrete ordinal variable (Low, Average, Moderate, High, Very High)
Has the person taking the action previously referred an action under the EPBC Act, or been responsible for undertaking an action referred under the EPBC Act?	Prior_referrals_for_proponent	Yes/No
Is there a record of Commonwealth environmental non-compliance?	Record_of_Cwth_noncompliance	Yes/No
Is there a record of other jurisdictional environmental non-compliance?	Record_of_other_noncompliance	Yes/No
Has the party to whom the decision will be granted ever been subject to any judicial proceedings under a Commonwealth, State or Territory law for the protection of the environment or the conservation and sustainable use of natural resources?	Judicial_proceedings_history	Yes/No
Primary location of action?	Project_Location	State/Territory of proposed action
EIS or EIA completed for other project components?	EIS_for_other_components	Yes/No
What is the main Sector relating to the action?	Subsector	Categorical sector variable
Proportion of MNES for which relative impacts of action are unlikely to be adequately addressed? ^(2)^	Percent_MNES_unaddressedimpact	Discrete interval variable (range 0–100)
**Consequence of project non-compliance**		
What is the current state of the environment in the proposed action location?	Current_state_of_Environment	Ordinal variable (Unmodified pristine, Partly modified, Highly modified, Previously developed)
Does the proposed project pose a significant risk to EPBC vulnerable listed threatened species?	Vulnerable_spp_risk	Yes/No
Does the proposed project pose a significant risk to EPBC endangered listed threatened species?	Endangered_spp_risk	Yes/No
Does the proposed project pose a significant risk to EPBC critically endangered listed threatened species?	Critically_endangered_spp_risk	Yes/No
Does the proposed project pose a significant risk to EPBC extinct in the wild listed threatened species?	Extinct_in_wild_spp_risk	Yes/No
Does the proposed project pose a significant risk to EPBC endangered listed threatened ecological community?	Endangered_TECs_risk	Yes/No
Does the proposed project pose a significant risk to EPBC critically endangered listed threatened ecological community?	Crit_endangered_TEC_risk	Yes/No
Does the proposed project pose a significant risk to EPBC listed migratory species?	Migratory_spp_risk	Yes/No
Does the proposed project pose a significant risk to World heritage properties?	World_heritage_risk	Yes/No
Does the proposed project pose a significant risk to National heritage places?	National_heritage_risk	Yes/No
Does the proposed project pose a significant risk to Wetlands of international importance?	Wetlands_intl_importance_risk	Yes/No
Does the proposed project pose a significant risk to the Commonwealth marine environment?	Cwlth_marine_environ_impacts	Yes/No
Does the proposed project pose a significant risk to the Great Barrier Reef marine park?	GBR_marine_park_impacts	Yes/No
Does the proposed project include nuclear actions?	Nuclear_action	Yes/No
Does the proposed project pose a significant risk to a water resource, in relation to a coal seam gas development or coal mining development?	Water_impacts_CSG_or_Coal	Yes/No
Does the proposed project pose a significant risk to any other Commonwealth controlling provision?	Controlling_provisions_risk	Yes/No
What is the expected significance of the impact on the MNES if project is non-compliant? ^(3)^	Significant_impact_risk	Ordinal variable (No impact, Low, Medium, High)

^(1)^ At the time that research was conducted, the DoE used total project cost as a proxy for project complexity. Projects with total cost <$40,000 = Low complexity; $40,001–100,000 = Average complexity; $100,001-$300,000 = Moderate complexity; $300,001–1,000,000 = High complexity; and >$1,000,001 = Very high complexity.

^(2)^ The proportion of MNES for which the relative impacts of action are unlikely to be adequately addressed is computed based on the proportion of MNES present for which there is expected to be an impact resulting from non-compliance (No impact/impact) that is not managed by an impact management strategy.

^(3)^ Significance of impact on MNES is assessed based on the expected impact of non-compliance (No impact, Low, Medium, High) and whether there is an impact management strategy to mitigate possible impacts (Yes/No) for each MNES. The overall significance of the impact is computed using the maximum expected impact of non-compliance (across all MNES) for which there is no impact management strategy; i.e. the significance of the impact is the worst expected unmanaged outcome for any individual MNES.

To test the feasibility of using the BBNs, we investigated two questions: how much data do we need to accurately predict risk using the naïve BBNs; and how sensitive is the risk outcome to different indicators? We describe the methods used for each question in the following subsections.

### How much data do we need?

Improving the DoE’s capacity to predict risk is likely to require collecting data on risk factors. However, prior to this project, the DoE did not use a quantitative risk-based approach during the assessment process and did not have a historical database of the chosen risk factors (although some of the required information may be collected, it is not currently in a single accessible database). Therefore, data from actual projects were not available for this study. Data collection is resource-intensive and the expense should be shown to be beneficial before data collection commences. A goal of this paper was to justify future data collection by determining whether a risk-based approach was feasible, and quantify the amount of data required to obtain output that was sufficiently accurate to be used by the DoE. To do this, we used simulated data.

**Fig 3:**
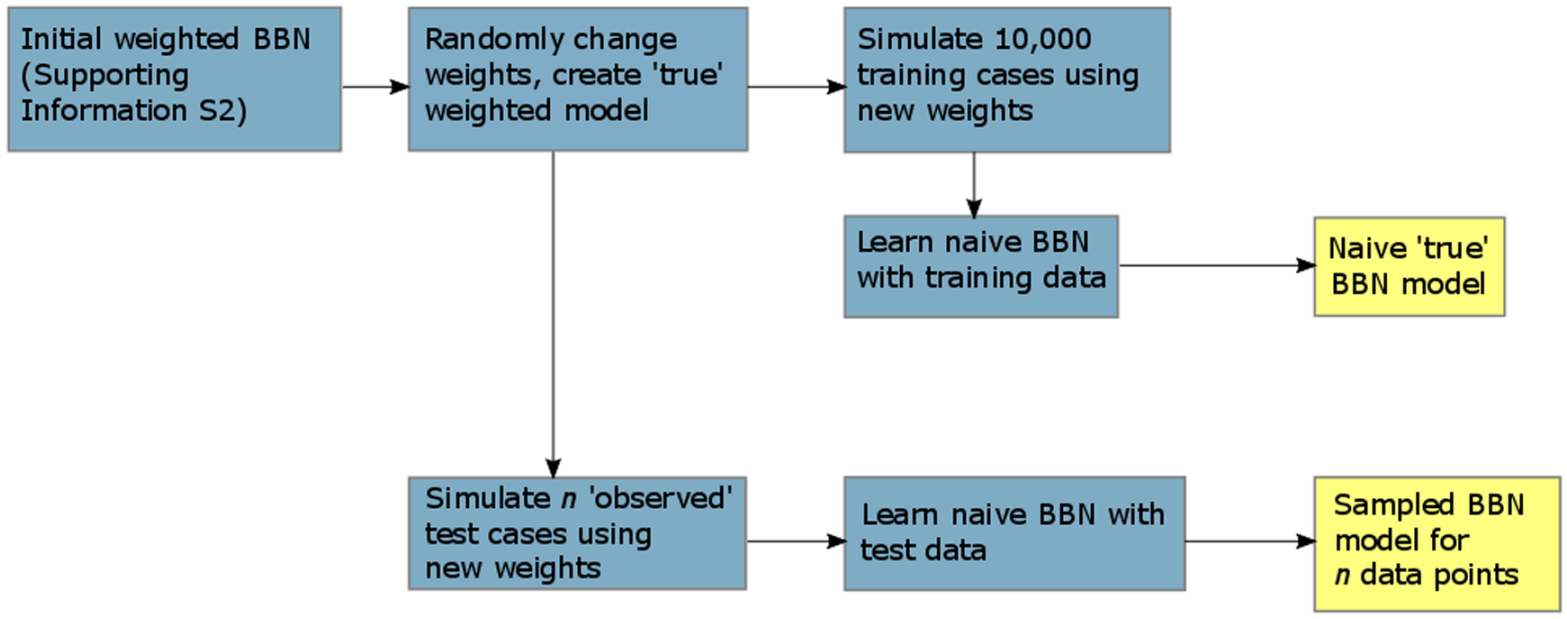
Simulation procedure for determining the learning ability of the BBN. For a given combination of weights (‘true’ model), we compared the ability of the BBN to learn the ‘true’ model given different amounts of data (sampled BBN model; we used *n* = 1, 100, 500, 1000 and 5000 test cases). The simulation procedure is repeated for 10 sets of randomly selected weights.

We used a simulation procedure to test the learning ability of the BBNs (Fig 3; Matlab and Netica-J code is included with S5 File). The procedure is based on the idea that there are a set of true weights that represent the actual contribution of each indicator to risk (note that the true weights may differ from the expert-derived weights used in the risk calculator if the experts did not predict the weights perfectly). We do not know the values of these true weights, but we expect to learn them given sufficient data.

Although we do not know the values of the true weights, we can assume some ‘true’ weights for the purposes of determining the amount of data required for accurate prediction. Our performance testing has three main steps. Firstly, we use the assumed weights to generate a naïve BBN representation of the assumed-weight ‘true’ BBN. For the purposes of the simulation, this step generates a risk model with weights that represent the exact contribution of each indicator to the risk outcome. Secondly, we draw fixed-size samples of data from the naïve ‘true’ BBN and learn a BBN from this sample data. This process simulates the expected outcome of predicting the ‘true’ BBN with only a limited number of observed projects. Finally, we use the sample BBN to predict the behaviour of the ‘true’ BBN model and quantify the prediction accuracy. This final step quantifies how well we expect to predict risk using a sample of observed projects. We repeat this process for different samples and for different values of assumed weights to obtain a distribution of predicted performance for an observed sample of given size. The remainder of this section provides details of the three steps in the simulation process.

Firstly, to generate a naïve BBN from the assumed weights, we change the values of the weights in the weighted BBN, and generate a large number of training data cases using this ‘true’ weighted distribution (cases are generated automatically using Netica’s “Simulate Cases” tool). With a large enough data set we can learn the exact naïve Bayes net representation of the ‘true’ model. We obtained a large data set by simulating 10,000 cases for each set of weights that we considered. These 10,000 cases are then used to train a naïve Bayes net representation of the network (see previous section and S3 File), which we refer to as the naïve ‘true’ BBN. The performance of the naïve ‘true’ BBN was assessed manually to confirm that it behaved identically to the ‘true’ weighted network.

Secondly, to generate fixed-size samples of ‘observed’ data, we used the “Simulate Cases” function in Netica to simulate a fixed-size sample of test data cases from the naïve ‘true’ BBN. We used this limited test data set to train a naïve BBN representation of the network. It returns a sampled BBN with the same structure as the naïve ‘true’ BBN, however because it was trained with limited data, it predicts different probabilities of non-compliance.

Finally, the performance of the sampled BBN model was compared to the ‘true’ model using the root-mean squared deviance (RMSD) and error rate. Because there were fewer data cases in the sampled test set than the 10,000 cases in the training set, we expected that the sampled model would not be as accurate as the ‘true’ model. We expected that as the amount of sample data increases, the performance of the sampled BBN will approach that of the naïve ‘true’ BBN.

We repeated this simulation approach 20 times each for sampled ‘observed’ data sets of size 1, 100, 500, 1000 and 5000 data points. After completion we re-ran the analysis, selecting a new set of ‘true’ weights to remove the bias associated with choosing a particular set of ‘true’ weights (we repeated this approach for 10 different values of the weights).

### Measuring the predictive power of the BBN

We measured the predictive power of the sampled BBN in two ways. Firstly, we calculated the root-mean squared deviance (RMSD) to determine how well the sampled BBN predicts the ‘true’ BBN for different amounts of data. Secondly, we used the error rate to determine how the number of misclassifications is likely to change given the amount of data and the risk tolerance of the decision-maker [45]. We used the Netica-J APIs and Matlab R2012a to program these analyses; Matlab code is provided with S5 File.

The RMSD is a measure of the average difference between predicted and observed estimates [46]. For *N* data points, RMSD is calculated using the following formula:

$$RMSD=\;\sqrt{\frac{\sum_{N}{\left(obs-pred\right)}^{2}}{N}}$$ (1)

Where *obs* is the observed risk score using the sampled BBN and *pred* is the predicted risk score using the ‘true’ BBN. The units of RMSD are the same as the units of risk (i.e. a 0–1 normalized value). By measuring the RMSD with sampled data sets of different sizes we can obtain the expected error of the BBN for a given amount of data.

Although the RMSD results will demonstrate how well the BBN can be learned with a fixed number of data points, they will not tell us how the decision-maker’s attitude to risk can contribute to errors. The output of the BBN is a probability between 0 and 1, and the decision-maker needs to use this to determine whether or not the risk will be realized. For example, if the decision-maker decides that all projects with a risk greater than a threshold value (e.g. 0.5) will be investigated, there are four possible outcomes:

1. The project has a predicted risk greater than the threshold, and risk is realized. The decision-maker made the correct decision to investigate this high-risk project.
2. The project has a predicted risk greater than the threshold, but the risk is not realized. The decision-maker did not need to investigate this project and has wasted resources (Type I error).
3. The project has a predicted risk less than the threshold, and risk is realized. The decision-maker failed to investigate and the risk was realised—this result can have serious consequences (Type II error).
4. The project has a predicted risk less than the threshold, and risk is not realized. The decision-maker made the correct decision not to investigate this low-risk project.

The relative frequency of these four outcomes depends on the decision-maker’s attitude to risk. Decision-makers that are risk-averse will choose to investigate all projects with a predicted risk greater than a low tolerance value (e.g. investigate all projects with risk >0.2). This is likely to reduce the number of type II errors, but will cost more because more projects will require investigation (increasing the number of type I errors). Conversely, a risk-seeking decision-maker will choose to investigate all projects with a predicted risk greater than a high tolerance value (e.g. investigate only projects with risk >0.8). For a given threshold, a confusion matrix shows the number of cases in each of the four kinds of risk (Table 2).

**Table 2:** Confusion matrix illustrating the four types of decision outcomes. Type I and II errors are misclassifications. In Type I, resources are wasted acting on an event that did not occur; in Type II, the decision-maker failed to act on an event that did occur.

**Predicted outcome**	**Realised outcome**	
	Yes	No
Yes	Correct decision	Type I error
No	Type II error	Correct decision

The error rate measures the combined number of type I and II errors—this is the total proportion of misclassifications for a given risk tolerance. For a fixed set of cases, the error rate is a measure of the proportion of cases for which the sampled BBN incorrectly predicts that risks will/will not be incurred. As more data is collected, the error rate is expected to fall as the sampled BBN gains experience from data. An error curve shows the frequency of misclassified risk cases for different amounts of data.

### Sensitivity analysis

The relative influence of indicators on the risk outcome can be assessed using the mutual information. We obtained the mutual information for the likelihood BBN with expert-derived weights (Fig 1) using Netica’s Sensitivity to Findings tool [42]. Indicators that have high mutual information have greater influence on the risk score. Thus, mutual information can be used to inform data collection by focussing effort on reducing the uncertainty about indicators that have greater influence on the outcome [47].

In some cases, it is useful to assess how changes in the values of two risk indicators *f* ∈ *F*, *g* ∈ *G* influence the risk outcome (this is also known as the sensitivity to parameters). Formally, the change in probability Δ*P*_*fg*_(*q*)caused by changes in *f* and *g* given a set of findings *q* is given by:

$$\Delta P_{fg}\left(q\right)=P\left(q\right)-P\left(q,f,g\right)$$ (2)

This type of analysis is not readily available in Netica. To obtain the change in risk from two indicators that vary together, we learned the probability *P*(*q*, *f*, *g*) by simulating cases from the BBN using Netica, then exported the results to a spreadsheet, where we calculated Δ*P*_*fg*_.

## Results

### Predictive power of the BBN

**Fig 4:**
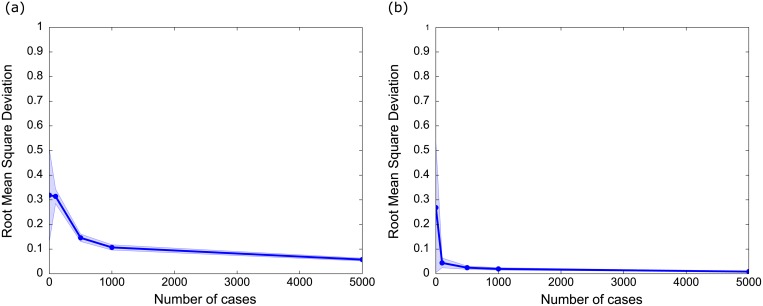
Simulated average root mean square deviation (RMSD) of the (a) likelihood and (b) consequence BBN for given numbers of training cases. The RMSD is the average difference in predicted likelihood between the naïve ‘true’ BBN and a BBN trained using a sample dataset containing the number of cases indicated on the x-axis. Larger training sets provide more information, making the BBN increasingly accurate as the number of cases increases. Error bars depict the mean standard deviation of simulated values.

Using only the prior weight information provided by experts (i.e. no training/sampled data), the average expected RMSD for both likelihood (Fig 4a) and consequence (Fig 4b) was high (~30%). On average, the risk predicted by the untrained sampled BBN would be ~30% different to the actual risk. However, as simulated data was added, the expected RMSD decreased rapidly. The consequence BBN was learned particularly rapidly—the RMSD was 4% with as few as 100 data points (Fig 4b). Learning was slower in the likelihood BBN; the RMSD did not drop to 10% until 1000 data points were collected (Fig 4a). The RMSD of the likelihood BBN was 15% with 500 data points. The likelihood BBN learns more slowly because some of the likelihood indicators have many possible categories (e.g. subsector, 22 categories; project location, 12 categories; and Percent MNES unaddressed, 10 categories), while the consequence BBN has few categories per indicator (maximum 4 categories).

In both likelihood and consequence BBNs, the standard deviation of the RMSD is high when there is no data, suggesting that the accuracy of BBN prediction varies substantially without data. However as data is added the standard deviation rapidly decreases in both BBNs, becoming negligible in both networks with greater than 500 cases.

Recall that the error rate is the proportion of misclassifications made by the learned BBN. A BBN can be very precise (i.e. predicts risk very precisely; low RMSD) but can misclassify whether or not the risk will be realized. The predicted outcome depends on the threshold of a ‘risky’ project, which is a policy decision. In our experiments we used a risk threshold of 50% (i.e. we predicted that risk would be realized if risk was >50%).

**Fig 5:**
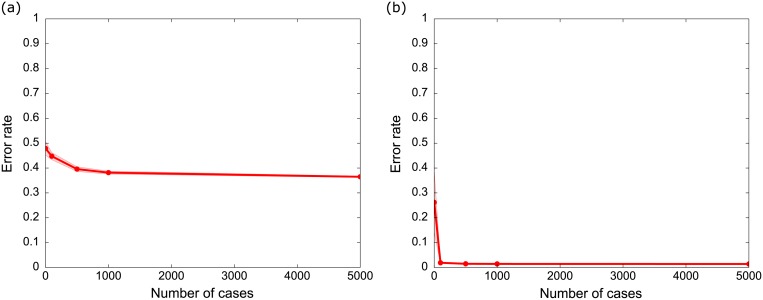
Error rate for the (a) likelihood and (b) consequence BBNs. Error rate is the proportion of misclassifications made by the learned BBN compared with 10000 data cases from the ‘true’ model. Error bars depict the mean standard deviation of simulated values.

With only the expert-based weights and no data, the BBN representation of the risk calculator had an error rate of 48% for likelihood and 27% for consequence (Fig 5). Although this may seem like poor performance, these simulations are compared to a randomly generated ‘true’ scenario. In reality, if the experts have a good understanding of risk, then the ‘true’ scenario is likely to be closer to the expert scenario and we would expect better predictive power from the BBN.

As data is added to the BBN, the error rate decreases. The decrease is dramatic for consequence, with an error rate of 2% with only 100 data points (Fig 5b). However the decrease is small for likelihood, and does not decrease below 36% even after 5000 data points are added (Fig 5a). This error rate is caused by the decision threshold and the properties of the likelihood BBN. The distributions of most indicators in the trained likelihood BBN are close to uniform; this means that when sampling this BBN to calculate the error rate, most samples will have a likelihood around 50%. Because the sampled likelihood is almost identical to the classification threshold, almost any variation in the sampled indicators will change the classification, even if the absolute change in likelihood is very small. Sampling will return many projects with likelihoods close to 50% that are predicted very precisely, but are misclassified and contribute to the error rate simply because they are close to the decision threshold. The apparently high error rates are an artefact of setting the risk threshold to 50%. Selecting either higher (e.g. consider risk realised if likelihood is >0.6) or lower risk threshold (e.g. consider risk realised if likelihood is >0.4) would result in a lower error rate because small changes in the risk score would not artificially change the classification of compliance or noncompliance.

### Sensitivity analysis and improving the predictive capacity of the BBN

In this section we describe the results of a sensitivity analysis applied to the likelihood BBN using the indicator weights provided by DoE staff. DoE staff chose to weight some indicators equally across all categories (e.g. project location and subsector) until data is collected that can provide more objective weightings. The lack of real data to set the weights means that our results demonstrate the kinds of analyses that are possible rather than provide real outcomes; without real data the sensitivity of the indicators cannot be truly known.

In our model, likelihood was most influenced by the risk factor “Record of Commonwealth non-compliance” and is not influenced by “Project location” (Table 3). Under current knowledge, users should focus on collecting information on a project’s “Record of Commonwealth non-compliance”, “Percentage of MNES unaddressed”, “Record of other non-compliance” and “Complexity score” rather than “Project location” to best estimate the risk outcome. Location is not expected to influence risk because the risk calculator applies the same score to all project locations. Data is needed to determine the contribution of this indicator to likelihood.

**Table 3:** Sensitivity to findings for the likelihood BBN. Risk factors with higher mutual information provide more information to the risk outcome.

Risk Factors	Mutual Information
Record of Commonwealth noncompliance	0.018
Percentage of MNES unaddressed	0.015
Record of other noncompliance	0.011
Complexity score	0.005
Prior referrals for proponent	0.005
Judicial proceedings history	0.004
Subsector	0.001
EIS for other components	0.001
Project location	0.000

**Fig 6:**
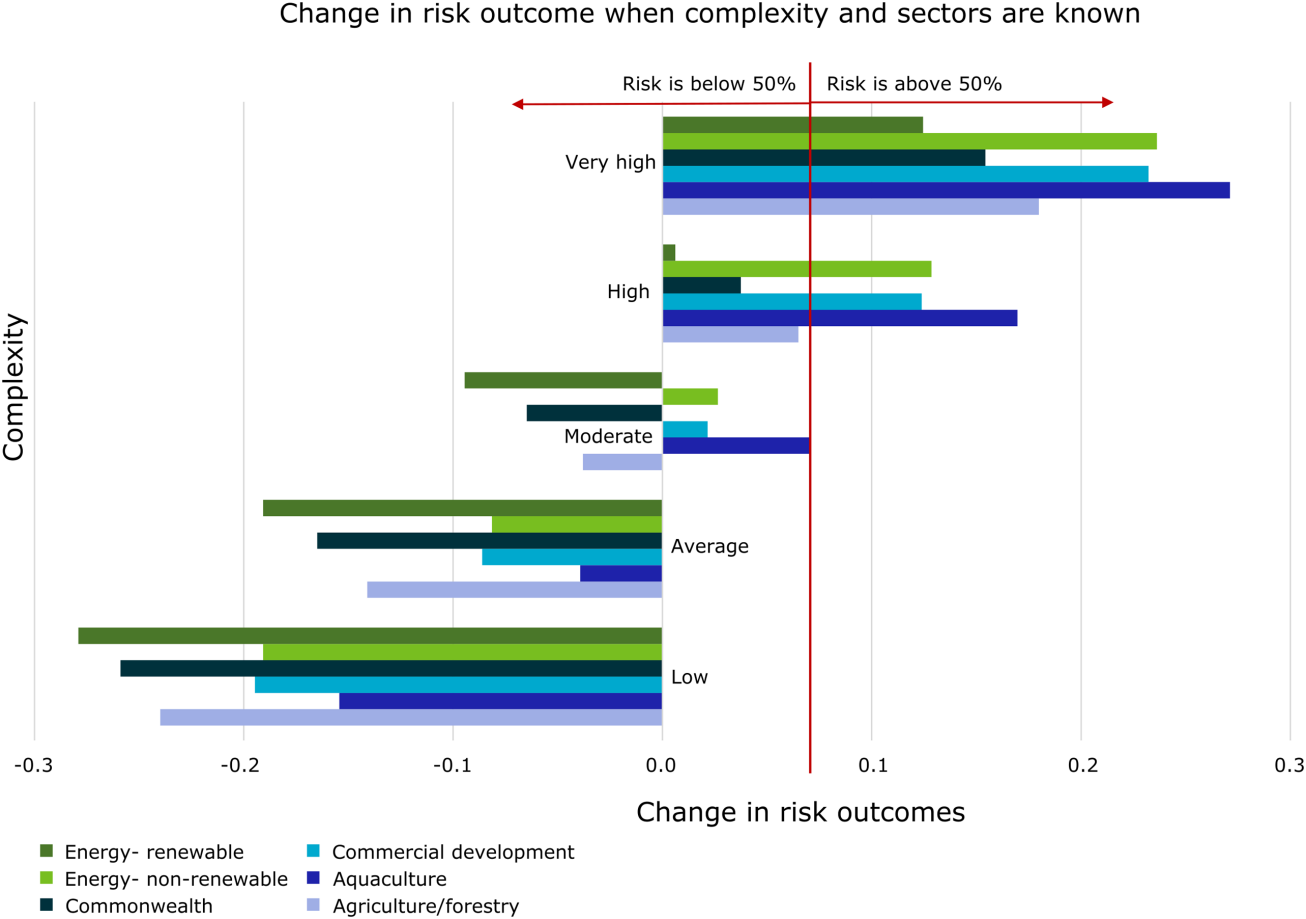
The sensitivity to parameters analysis shows how the risk factors ‘Complexity score' and 'Subsector' change the risk outcomes on average. The red line identifies a hypothetical decision threshold of 50% above which users may decide to apply additional scrutiny to a project. Some sectors are not shown for ease of viewing. The horizontal axis represents the change in risk outcomes compared to a project with no information (i.e., compared with the average risk value if we didn’t know the complexity or the sector risk factors; formally defined by Eq 2).

The sensitivity to findings obtained from Netica are calculated by varying only one indicator at a time. Users might be interested in the variation of risk outcomes for several risk indicators at a time. To illustrate this, we ran a sensitivity to parameter analysis on the two risk factors ‘Complexity score’ and ‘Subsector’ (Fig 6). This allows users to compare, for example, the relative risks associated with a low complexity non-renewable energy project and a high complexity renewable energy project. We created an illustrative scenario by generating simulated data with high weights on the risk factors ‘Complexity score’ and ‘Subsector’ (Fig 6). In the example, a high complexity is likely to increase the risk of a project by around 0.1 for most sectors (compared to a project with unknown sector and complexity). Conversely a low risk decreases the risk by around 0.15 for all sectors. We can also compare across sectors—for example, given these hypothetical weights, the increase in risk from a high complexity renewable energy project (+0.006) is much less than the increase in likelihood from a high complexity non-renewable energy project (+0.129).

The sensitivity to parameters can also inform how decisions will change if the actions are dependent on a particular decision threshold. Here, we plotted a hypothetical risk threshold of 0.5 (red line; Fig 6), i.e. if predicted likelihood was above 0.5, then the DoE would apply additional scrutiny to a project. In this figure, ‘very high’ and ‘high’ complexity projects have a risk greater than 0.5, with the exception of high complexity renewable energy sector projects. High and very high complexity projects in these sectors would be subject to additional scrutiny if this decision threshold was applied. Low-Moderate complexity projects from any sector shown in Fig 6 would not receive additional scrutiny based on this decision threshold, unless other risk indicators gave them above-average likelihood of non-compliance.

## Discussion

### Feasibility of predicting EIA risk with a BBN

Previous studies have stated that the effectiveness of EIA in predicting and preventing undesirable outcomes is largely unknown [7, 48]. However, experience from other disciplines suggests that risky outcomes can be learned given sufficient data [29, 49]. EIA is now used globally to manage the impacts of development projects on the environment [1], so there is an imperative to demonstrate its effectiveness; both to identify risky projects and reward good proponent behaviour. Although a further pilot test with real project data is still required, our results suggest that a BBN is a promising method to monitor risk within an existing EIA process given a modest investment in data collection. Our simulations showed that the BBN can determine risk for a randomly distributed set of unknown weights with 90% accuracy using approximately 1000 data points (Fig 4).

Our BBN approach is one of many classification algorithms that could be used to determine risk given a suitable data set; and the risk factors that were selected may not be generally applicable to all domains. However the algorithm and features used to classify risk may have little effect on predictive power; in many cases more data results in better prediction regardless of the model used [50, 51]. While this statement is not always correct, it is likely to be true if very little data exists; in our results the value of new data declines exponentially (Fig 4) so that even relatively small data sets may improve prediction. In essence, even if the data set and the model are not perfect, then any data is better for prediction than none. To our knowledge, no EIA agencies currently collect information on risk factors with the goal of predicting noncompliance of future development projects in a structured way. Although data collection costs time and resources for resource-constrained agencies, our study suggests that there may be substantial gains in predictive power using relatively few data points. If this is a general result, EIA agencies that do not collect data on risk factors may consider instituting formal tools to collect data on risk factors as part of their routine operations.

### Using the BBN

Given that risk can be predicted precisely with a BBN approach, what are the potential applications of a risk-based model? An obvious application is to aid decision-making for EIA. In principle, the risk model could be used to guide decisions directly, so that high risk projects would be accepted, conditioned or refused based on BBN predictions. However this approach may not be applicable in practice, as EIA decision-making must balance both the costs and benefits of development projects on the environment, so the risk of environmental harm is only one aspect of a decision that must also incorporate many other complex societal values [52]. The ability to use a risk-based approach within existing legislative frameworks is also unclear and would need to be debated in each jurisdiction. Despite these caveats, the tool may be useful as a decision-aiding tool that summarises large amounts of information about the likely causes of risky behaviour in an objective way, allowing the decision-maker to allocate their limited resources in a way that minimises the likelihood of adverse outcomes.

A simple way to use the BBN would be to apply additional scrutiny to projects with a risk greater than a fixed risk threshold (e.g. investigate all projects with risk greater than a threshold), however care needs to be taken when selecting this threshold to avoid either wasting resources over-scrutinising projects that are unlikely to offend (risk threshold set too low); or failing to scrutinise projects that will offend (risk threshold too high) (Fig 5). The trade-off involved in selecting this risk preference can be optimised using multi-objective optimisation techniques [53]. These techniques seek a compromise solution that minimises one or both kinds of errors while maximising the number of correct classifications.

The tool could also be used for resource allocation within an EIA agency. Given a finite resource budget and a fixed risk threshold, a list of candidate projects can be selected for additional scrutiny. A challenge is to determine the level of effort that should be spent scrutinising each project, and how to optimally schedule this effort across the project portfolio over time. An optimal schedule would minimise the overall risk over a portfolio of projects over time, given a limited resource budget. This is more difficult than selecting only the highest-risk projects. For example, the risk scores for projects are unlikely to change over time once approval is granted. An optimal workflow would need to include a dynamic component that accounted for the time since the last compliance investigation. Similarly, if only high-risk projects were selected for scrutiny, then proponents of medium- and low-risk projects may soon learn that they were very unlikely to be scrutinised. A categorical allocation of effort may be the best way to address this issue; i.e. allocating a proportion of total effort to low and medium risk projects. Some effort would also need to be allocated to random investigations to ensure that the system was difficult to predict for proponents.

Another key use for the BBN is to quantify the relative importance of risk factors using sensitivity analysis. This has two main advantages. Firstly, the relative importance of individual indicators can be easily assessed (Table 3), which can be used to refine the BBN. In this study, the number of possible combinations of risk factors for both likelihood (1.27 million possible combinations) and consequence (1.04 million combinations) is very large. Although the BBN can predict the outcomes of a combination of risk factors that it has not encountered, it will learn more rapidly if there are fewer combinations. Once a subset of data has been collected, the BBN may be improved by determining which risk factors contribute significantly to likelihood and consequence (see Table 3), and removing those which are found to have minimal influence. Similarly, reducing unnecessary categories within a risk factor can greatly reduce the number of risk factor combinations. Removing a single category reduces the number of combinations to at least half of the original number of combinations, so it is worth scrutinising each category to determine whether it is necessary to predict risk.

Secondly, the sensitivity of combinations of risk factors can be assessed using the BBN (Fig 6). This can be used to direct resources to key risk areas. For example, if projects from a subsector in a location were found to have a higher likelihood of noncompliance than projects from that subsector in other locations, then resources could be directed to improve compliance in that location. Improvements can be tracked over time using the change in risk as a result of the increased resources. This kind of reasoning is achievable because the BBN maps causal dependencies and represents them as probabilities—the output is interpretable and the cause of changes in risk can be attributed directly [19].

### Continual learning

An advantage of the BBN approach is that it allows for continual learning to occur [21]. While all organisations are likely to learn from experience, many do so informally so that the knowledge gained is stored as experience for staff members and not captured formally. This may create a liability for organisations with high staff turnover, because staff may leave, taking their knowledge with them [8]. In the BBN approach, existing staff knowledge can be combined with data to create a prediction tool that can be used immediately. Subsequently, every assessed project that is subsequently checked for compliance creates a feedback data point for the BBN that contributes to improved risk prediction. This approach ensures that every project contributes to learning; ensuring that the organisation has a structured process to capture knowledge.

If the tool is to be implemented immediately and ‘learn by doing’ as data is collected, a decision needs to be made about how much weight to give to initial weights obtained from experts relative to data. In our simulations we gave the expert weights (prior distribution) the same importance as a data point, so the elicited weights had little influence on the results. However the importance of the elicited data can be increased if required. This will mean that the BBN takes longer to learn, but it will be more stable and make more consistent decisions, particularly when only a little data has been added to the BBN. Note that while rapid learning can be desirable, there can be a downside if the BBN recommends changing the outcome decision too often, for example if the same set of risk factors lead to different outcomes within a short space of time. Similarly, consistency can be desirable in some circumstances, but if the BBN recommends the wrong decision consistently, this is unlikely to be desirable. The relative importance of the initial weights is a trade-off between the need for consistency from the BBN and the need to learn as rapidly as possible. There was little value in investigating this without real data. However once a subset of data has been collected, this issue could be investigated using different weights to find a suitable trade-off.

### Contribution to BBN techniques and practice

Applying the BBN to the field of EIA risk prediction for development projects resulted in the development of two techniques that may be used in other fields.

Firstly, our study provides a method to assess the number of BBN data points required to predict with an acceptable accuracy level before data collection commences. This is useful in applications where collecting data is often expensive and/or time-consuming and the amount of data required may be a major practical constraint to BBN application. After running the simulations, if the data requirements are deemed excessive, the BBN can be simplified or a different approach can be investigated.

Secondly, our method to convert a weighted-sum risk tool into a naïve BBN creates the opportunity for continuous learning from new data in systems currently managed using weighted-sum risk tools (S3 File). Weighted-sum risk calculators are very common in risk prediction because of their simplicity, but they have drawbacks (see Introduction), including that they cannot adapt to new data. Although methods exist to convert weighted-sum calculations into BBNs [19], the resulting BBNs have an unobservable ‘weightings’ node which adapts to findings, but does not learn from new data. Converting the weighted BBN into a naïve BBN removes the need for the unobservable node, allowing continuous learning to occur. The approach that we applied to convert the weighted BBN to a naïve BBN is easily performed using built-in functions in Netica and is transferrable to other applications that use a weighted-sum risk approach.

## Conclusions

Although overall risk-based approaches are well-developed for a number of related environmental fields, to our knowledge, there are few formal quantitative systems to learn the risks posed by construction and development projects requiring EIA. This is surprising, given that EIA is often the primary method for controlling the impacts of development projects on the environment. EIA assessments often consider potentially severe or irreversible consequences where the impacts of development projects are highly uncertain, so there is a demand for objective, data-driven information. A risk-based approach has many potential advantages, e.g. improved prediction and attribution of risk; sensitivity analysis; continual learning; and optimal resource allocation. Here we demonstrate that established data mining techniques provide the necessary tools to represent overall risk for EIA, and that our approach is feasible given sufficient data. Although further testing with real data is desirable to provide certainty about the effectiveness of a BBN to predict overall EIA risk, other disciplines are already using BBNs to manage analogous problems, so there is a strong precedent for applying these techniques to an EIA context. In many cases the only technical barrier to entry is data availability, so we suggest that regulators who may anticipate using an overall risk-based approach in future should begin to collect data as early as possible.

## Supporting information

S1 File Overview of EPBC Act EIA process. (DOCX)

S2 File List of indicators and weighted BBNs. (DOCX)

S3 File Steps to create a naïve BBN from the weighted sum approach. (DOCX)

S4 File Weighted BBN Netica files. (ZIP)

S5 File Matlab code for analysis. (ZIP)
