# Supplementary material for: A preliminary approach to quantifying the overall environmental risks posed by development projects during environmental impact assessment
Source: PLoS One. 2017 Jul 7;12(7):e0180982. doi: 10.1371/journal.pone.0180982 (PMC5501652; doi:10.1371/journal.pone.0180982)
Supplement: S1 File — (DOCX) [file pone.0180982.s001.docx]

# Supporting Information: Quantifying Risk in Environmental Impact Assessment

# S1: Overview of EPBC Act EIA Process

The Australian Department of the Environment oversee and regulate actions that have the potential to have a significant impact on a matter of national environmental significance (MNES). There are nine MNES that are protected under the EPBC Act: world heritage properties, national heritage places, wetlands of international importance (Ramsar sites), listed threatened species and ecological communities, migratory species, Commonwealth marine areas, the Great Barrier Reef Marine Park, nuclear actions, and water resources (in relation to coal seam gas development and large coal mining development).

The EPBC Act sets the statutory timeframes, decision points, and matters that must be taken into account by the decision maker in order to approve the taking of an action. The assessment and approval process set out in the EPBC Act and related policy documents can be broken down into four main stages (see Figure 1).

Once the initial decision that the proposed action is a controlled action (EPBC Act applies) has been made, the primary aim of the assessment process is to ensure that there is a high level of certainty about the impacts of the proposed action on MNES, and any measures required to address those impacts (avoid, mitigate, offset) are identified and agreed by all parties prior to an approval decision being made. For the purposes of this manuscript, the risk approach provides an assessment of risk associated with non-compliance with the approval decision (post-assessment, Stage 4).

**Post-approval compliance and monitoring**

**Approval decision and condition setting**

**Controlled Action decision**

Figure 1: Summary of stages of EPBC assessment and approval processes. In this manuscript, risk scores are calculated at stage 4 of the process.
