# Supplementary material for: A preliminary approach to quantifying the overall environmental risks posed by development projects during environmental impact assessment
Source: PLoS One. 2017 Jul 7;12(7):e0180982. doi: 10.1371/journal.pone.0180982 (PMC5501652; doi:10.1371/journal.pone.0180982)
Supplement: S2 File — (DOCX) [file pone.0180982.s002.docx]

# Supporting Information: Quantifying Risk in Environmental Impact Assessment

# S2: List of Indicators and weighted BBNs

## List of indicators used in BBN

This section contains a list of the indicators used in the likelihood and consequence BBNs. Further information on the indicators, including the initial weights, is contained in [Nicol *et al.* (2015](#_ENREF_24)). Initial weights are also shown in the ‘weights’ node of Figures S1 and S2.

**Table 1: Consequence indicators used to predict project risk**

| Question | Node label |  |
| --- | --- | --- |
| What is the current state of the environment in the proposed action location? | Current_state_of_Environment | |
| How many of the following are MNES likely to be impacted by the proposed action and what is the expected maximum significance of impacts? |  | Maximum significance of impacts  (composite score gives Significant_impact_risk) |
| EPBC vulnerable listed threatened species? | Number_vulnerable_spp | No impact/Low/Medium/High |
| EPBC endangered listed threatened species? | Number_endangered_spp | No impact/Low/Medium/High |
| EPBC critically endangered listed threatened species? | Num_critically_endangered_spp | No impact/Low/Medium/High |
| EPBC extinct in the wild listed threatened species? | Num_extinct_in_wild_spp | No impact/Low/Medium/High |
| EPBC endangered listed threatened ecological community? | Number_endangered_TECs | No impact/Low/Medium/High |
| EPBC critically endangered listed threatened ecological community? | Num_critically_endangered_TECs | No impact/Low/Medium/High |
| EPBC listed migratory species? | Number_migratory_spp | No impact/Low/Medium/High |
| World heritage properties? | Number_world_heritage | No impact/Low/Medium/High |
| National heritage places? | Number_national_heritage | No impact/Low/Medium/High |
| Question | **Node label** |  |
| Wetlands of international importance? | Num_wetlands_intl_importance | No impact/Low/Medium/High |
| Commonwealth marine environment? | Cwlth_marine_environ_impacts | No impact/Low/Medium/High |
| Great Barrier Reef marine park? | GBR_marine_park_impacts | No impact/Low/Medium/High |
| Nuclear actions? | Nuclear_action | No impact/Low/Medium/High |
| A water resource, in relation to a coal seam gas development or coal mining development? | Water_impacts_CSG_or_Coal | No impact/Low/Medium/High |
| Other Commonwealth controlling provision? | Num_pot_controlling_provisions | No impact/Low/Medium/High |

Table 2: Likelihood indicators used to predict project risk

| Question | Node Label |
| --- | --- |
| What is the relative complexity score based on total fee estimate? | Complexity_score |
| Has the person taking the action previously referred an action under the EPBC Act, or been responsible for undertaking an action referred under the EPBC Act? | Prior_referrals_for_proponent |
| Is there a record of Commonwealth environmental non-compliance? | Record_of_Cwth_noncompliance |
| Is there a record of other jurisdictional environmental non-compliance? | Record_of_other_noncompliance |
| Has the party to whom the decision will be granted ever been subject to any judicial proceedings under a Commonwealth, State or Territory law for the protection of the environment or the conservation and sustainable use of natural resources? | Judicial_proceedings_history |
| Primary location of action? | Project_Location |
| EIS or EIA completed for other project components? | EIS_for_other_components |
| What is the main Sector relating to the action? | Subsector |
| Proportion of MNES for which relative impacts of action are unlikely to be adequately addressed? | Percent_MNES_unaddressedimpact |

## BBNs converted from risk calculator

Figure 2 and Figure 3 are the most direct representations of the risk calculator that can be created using the BBN framework. Weights elicited from DoE staff are contained in the node labelled “weights”. Due to the number of categories in these BBNs and the structure imposed by the weights, there are a very large number of possible combinations of risk factors in these BBNs. To represent the full risk calculator as a network, we added intermediate nodes (grey nodes), which are used as summary nodes and reduce the number of combinations that must be considered by any child node. We removed these intermediate nodes and the need for weights by representing Figure 2 and Figure 3 as naïve BBNs (see main text).


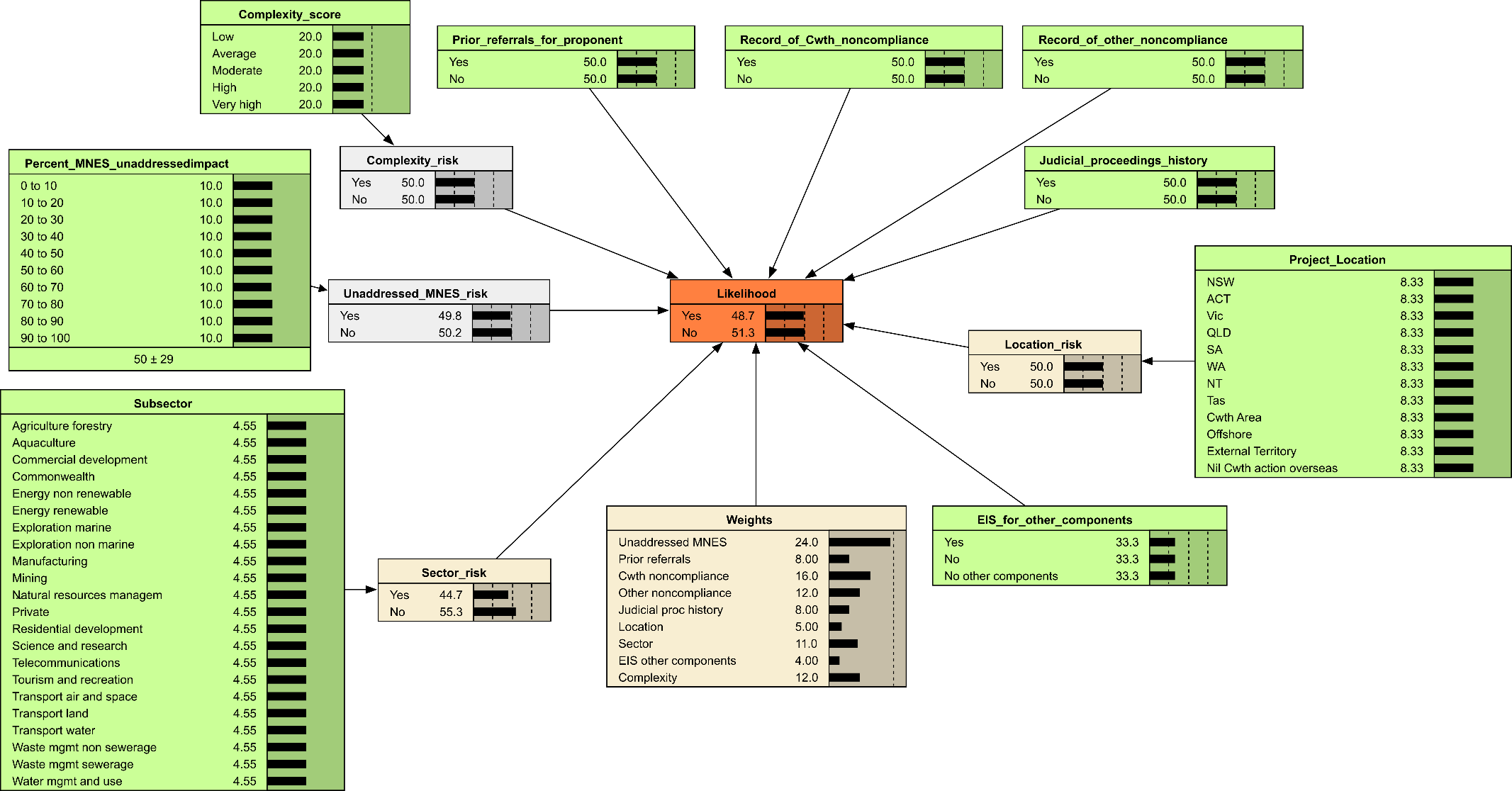


Figure 2: Likelihood BBN translated directly from the NESTRA risk calculator.


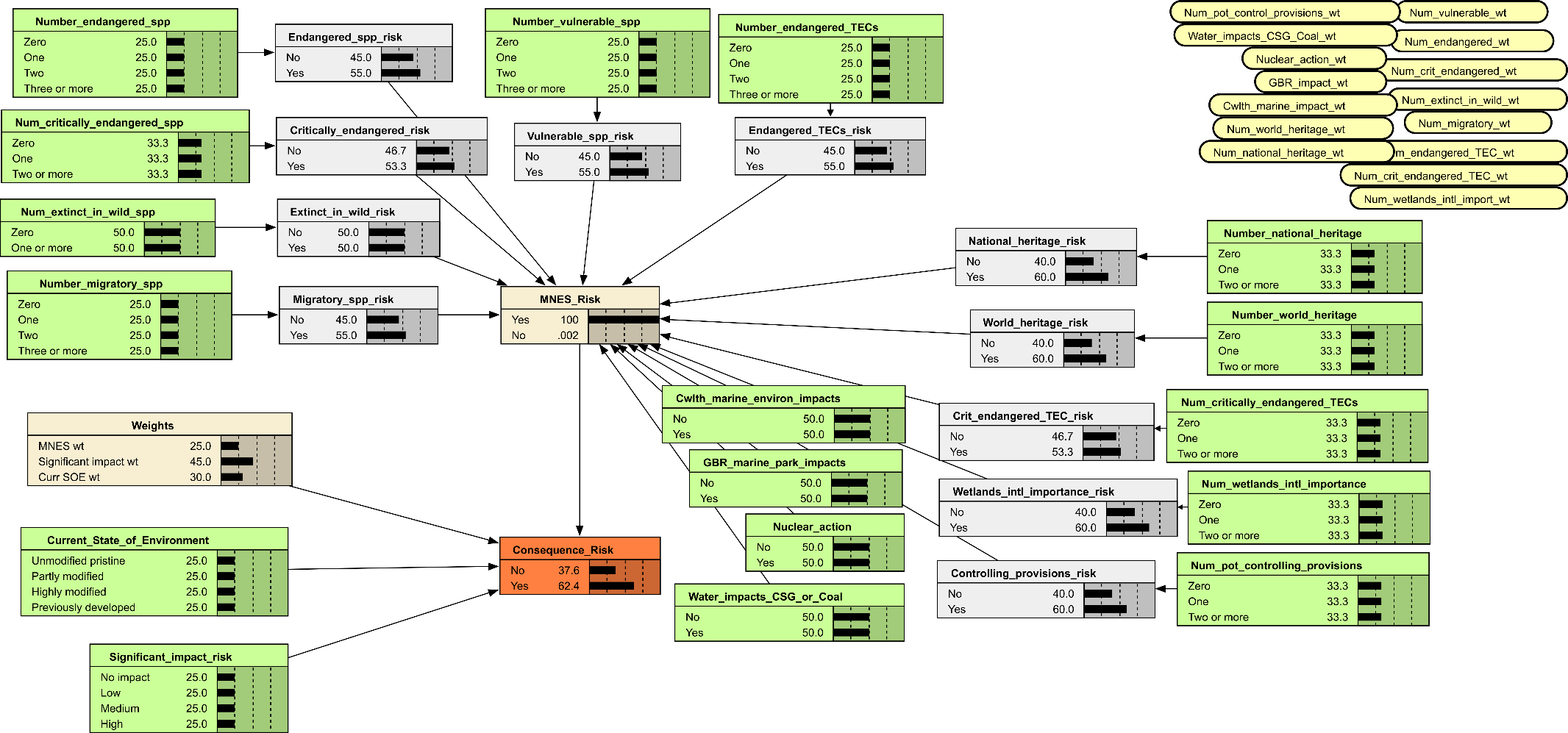


Figure 3: Consequence BBN translated directly from the NESTRA risk calculator
