# Supplementary material for: A preliminary approach to quantifying the overall environmental risks posed by development projects during environmental impact assessment
Source: PLoS One. 2017 Jul 7;12(7):e0180982. doi: 10.1371/journal.pone.0180982 (PMC5501652; doi:10.1371/journal.pone.0180982)
Supplement: S3 File — (DOCX) [file pone.0180982.s003.docx]

# Supporting Information: Quantifying Risk in Environmental Impact Assessment

# S3: Steps to create a naïve BBN from the weighted sum approach

This section provides instructions on how to convert the weighted sum approach to a naïve BBN using Netica. For further information on generating case files and learning, see the Netica help (<http://www.norsys.com/WebHelp/NETICA.htm>), particularly the chapters “Cases and Case Files” and “Learning from Case Files”

### Represent the weighted sum model as a BBN

We refer the reader to Fenton and Neil (2013), section 8.4.6, which outlines how to convert a weighted sum risk tool into a BBN. Our approach follows the Fenton and Neil methodology for this step.

To represent the weighted sum model as a BBN, create nodes for each risk factor, one node for the weights, and one node for the aggregate (likelihood or consequence) score (e.g. see Figure 2 of Supplementary information S2). Create links from each risk factor to the aggregate node, and a link from the weights to the aggregate node. Enter the expert-derived weights into the CPT for the weights node. For nodes with many states (or continuous states), you may need to create a summary node with a binary state to reduce the combinatorial complexity of the problem (e.g. see nodes “Complexity risk” and “Unaddressed MNES risk” in Figure 2 of Supplementary Information S2).

Once the structure of the net is created, we need to ensure that the risk factor nodes have the required influence on the aggregate score. We do this using equations in the aggregate score node. Open the aggregate node and select “Equation” in the dropdown box (default value shows “Description” in this box). The Netica equation syntax is somewhat complex; we recommend reading the “Equations” chapter in the Netica help documentation for further information. However for this approach most of the equations are binary and have similar syntax—mastering the syntax for one variable makes defining equations for the remaining variables relatively easy. The logic for a binary variable is: “if the risk factor occurs, then increase the aggregate score by the weight assigned to the risk factor”. For example, if the risk factor is “Record of other Non-compliance” (i.e. does the proponent have a history of non-compliance (Y/N)?), then the Netica equation syntax for this factor is:

P (Likelihood |Other_noncompliance, ….)=

Weights== Other_noncompliance ? (

Record_of_other_noncompliance== Yes ? 1:0) :0

In plain English, this could be interpreted as: The likelihood probability depends on Other_noncompliance (and maybe other factors—enter all risk factors into the first line of the equation). If there is a record of other non-compliance, then add 100% of the weight associated with “Other_noncompliance” to the aggregate score; otherwise return a likelihood of 0.

These equations must be completed for each of the weights. Additional equations may be necessary if summary nodes are created to reduce the complexity of risk factor nodes with many states. We recommend looking at the equations in the Netica files included with the manuscript supplementary information for further guidance.

### Sample the weighted sum model to obtain case data

Our objective in this step is to sample a very large number of case data points from the weighted-sum BBN. Case data will be generated according to their probabilities of occurrence in the weighted-sum BBN. The principle is that if we sample enough case data, we can use the case data to build a BBN that performs in the same way as the weighted-sum BBN but has a different structure (i.e. without the unobservable weighting node). By removing the unobservable weighted node, we allow the new BBN to adapt to new data, removing the subjectivity of expert-derived weights that are not based on real data. Netica has a built-in function to sample data from a BBN. To sample case data from the weighted-sum BBN in Netica, select “Cases-> Simulate Cases”; specify 100000 cases in the pop-up box, and save the case file with an appropriate name. This will generate a text file containing 100000 case files that can be used to parameterize the naïve BBN.

### Build a naïve BBN with the desired structure

We now build a naïve BBN in which each risk factor directly influences the aggregate likelihood or consequence score without weights. The relative importance of risk factors will evolve from data as it is incorporated into the BBN. Naïve BBNs are relatively simple to construct: In a new Netica file, create all the observable nodes (i.e. the risk factors/indicators) from the weighted sum model, as well as the aggregate likelihood or consequence node. Do not create a node for the weights. Construct links from the aggregate node to each of the observable indicator nodes (e.g. See Figure 1 in the main text). Do not specify anything in the conditional probability tables for each node, as we will learn the conditional probability relationships using the case data generated by sampling from the weighted-sum BBN.

### Learn BBN probabilities with case data

Although we have created a naïve BBN with the desired structure, we still need to specify the probabilistic relationships between the aggregate score and each of the risk factor nodes. To do this and obtain an equivalent BBN to the original weighted-sum BBN, we need to learn from the case data sampled in step 2. Again, Netica provides a built-in way to do this. To learn from case data, select “Cases-> Learn -> Incorp Case File” and select the file that you saved in Step 2. Netica will then ask whether you want to remove existing node CPT and experience files first— answer “Yes”. Select a node degree of 1 at the next dialogue box, and Netica will automatically create conditional probabilities for each node. The resulting BBN should now operate in the same way as the weighted-sum BBN. Test a few combinations to make sure that this is the case. If the BBN does not operate identically to the weighted-sum BBN and no other errors have occurred, sample more states in the case file and re-learn the naïve BBN.
